# Roles of the *RET *Proto-oncogene in Cancer and Development

**DOI:** 10.31662/jmaj.2020-0021

**Published:** 2020-07-07

**Authors:** Masahide Takahashi, Kumi Kawai, Naoya Asai

**Affiliations:** 1 Department of Pathology, Nagoya University Graduate School of Medicine, Nagoya, Japan; 2 International Center for Cell and Gene Therapy, Fujita Health University, Toyoake, Japan; 3 Department of Pathology, Fujita Health University, Toyoake, Japan

**Keywords:** *RET*, oncogene, DNA rearrangement, Thyroid Cancer, Lung Cancer, Multiple Endocrine Neoplasia type 2, Hirschsprung’s Disease, Development

## Abstract

*RET* (*REarranged during Transfection*)**is activated by DNA rearrangement of the 3′ fragment of the receptor tyrosine kinase gene, namely, *RET *proto-oncogene, with the 5′ fragment of various genes with putative dimerization domains, such as a coiled coil domain, that are necessary for constitutive activation. *RET *rearrangements have been detected in a variety of human cancers, including thyroid, lung, colorectal, breast, and salivary gland cancers. Moreover, point mutations in* RET* are responsible for multiple endocrine neoplasia types 2A and 2B, which can develop into medullary thyroid cancer and pheochromocytoma. Substantial effort is currently being exerted in developing *RET *kinase inhibitors. *RET* is also responsible for Hirschsprung’s disease, a developmental abnormality in the enteric nervous system. Gene knockout studies have demonstrated that *RET *plays essential roles in the development of the enteric nervous system and kidney as well as in spermatogenesis. Studies regarding *RET *continue to provide fascinating challenges in the fields of cancer research, neuroscience, and developmental biology.

## Structure and Expression of the *RET* Proto-oncogene

**Figure 1.**
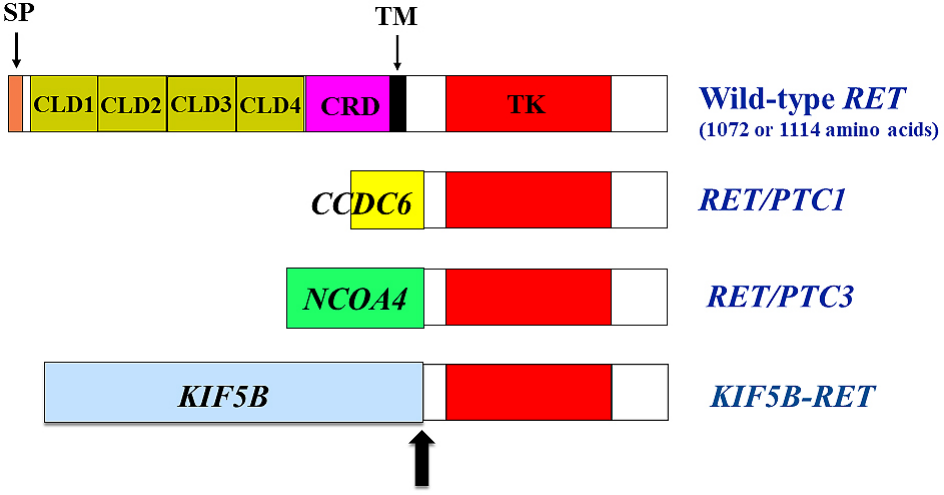
*RET* rearrangement is detected in papillary thyroid cancer (PTC) and non-small cell lung cancer (NSCLC). *RET/PTC1* and *RET/PTC3 *were reported to be detected in 5%–35% of adult and 60%–70% of childhood PTCs after the Chernobyl reactor accident, respectively. *KIF5B-RET* was identified mainly in NSCLC. SP, signal peptide; CLD, cadherin-like domain; CRD, cysteine-rich domain; TM, transmembrane domain; TK, tyrosine kinase domain.

The *RET *oncogene was first identified from the transfection of NIH3T3 cells in 1985 ^(1)^. *RET* proto-oncogene encodes a receptor tyrosine kinase and is primarily expressed as two isoforms of 1072 (short isoform) and 1114 (long isoform) amino acids by alternative splicing in the 3′ region ^(2)^. Overall, 51 carboxyl-terminal amino acids in the long isoform are replaced by nine unrelated amino acids in the short isoform. The presence of four repeated cadherin-like domains in the RET extracellular domain is unique among receptor tyrosine kinases (Figure 1) ^(3)^. As observed for cadherins, the binding of Ca^2+^ ions in the cadherin-like domain is necessary for RET activation ^(4)^. The extracellular domain also contains a cysteine-rich region with 16 cysteine residues ^(5)^, six of which are mutated in MEN2A families.

Analyses using in situ hybridization and immunohistochemistry have identified RET mRNA and protein expression in restricted tissues and cells during embryogenesis and after birth ^(6), (7)^. During embryogenesis, RET expression is detected in the excretory system, including the nephric duct, ureteric bud, and collecting ducts of the kidney. In the developing peripheral nervous systems, it is highly expressed in enteric neural crest cells and autonomic and dorsal root ganglia. In the central nervous system, RET protein is expressed in the neuroepithelial cells of the ventral neural tube as well as in several cranial ganglia, including facial, glossopharyngeal, trigeminal, and vagus cranial ganglia. After birth, neurons in the nervous systems mentioned above continue to express RET protein, whereas its expression has not been observed in the kidneys of adult rats.

## Identification of the RET Ligands

In 1996, although GDNF does not bind to RET directly, it was found that glial cell line-derived neurotrophic factor (GDNF) could activate RET kinase. A glycosylphosphatidylinositol-anchored cell surface protein, GDNF family receptor α1 (GFR α1), is necessary for GDNF binding, and the GDNF-GFR α1 complex mediates RET dimerization ^(8), (9)^. Subsequently, three further GDNF family ligands (GFLs), including neurturin (NRTN), artemin (ARTN), and persephin, were identified and found to bind to GFR α1, α2, and α3, respectively, leading to RET activation ^(10), (11)^. GFRα family members contain three globular cysteine-rich domains (D1, D2, and D3) except for GFRα4, which lacks the N-terminal D1. GDNF functions as a homodimer and binds to the D2 domain of GFRα1^(11)^. The structure of the GFL-GFRα complex is common among the four GFL-GFRα pairs. When two molecules of RET are recruited to the lipid raft by the GDNF-GFRα1 dimeric complex, the two cysteine-rich domains of RET are brought into close proximity, thus promoting dimer formation and the activation of tyrosine kinase ^(11)^. Recent cryo-electronic microscopic analysis has determined the structure of the NRTN-GFRα2-RET complex ^(12)^. The reconstitution of the extracellular areas of the NRTN- and GFRα2-bound RET signaling complex revealed that the RET extracellular domain interacts with the NRTN-GFRα2 complex via a large surface area. During this interaction, RET cadherin-like domains 1–3 interact with the GFRα2 D2 and D3 domains, and the unmodeled RET cysteine-rich domain is in contact with NRTN and GFRα2. This explains how complex formation leads to RET receptor dimerization. Despite large interaction surfaces, the GFRα2 and RET extracellular domains do not appear to be associated in the absence of NRTN.

Gene knockout (KO) mice for GDNF, GFRα1, or RET die soon after birth and share morphologically similar phenotypes, including a lack of enteric neurons in the entire enteric nervous system, kidney agenesis or severe dysgenesis, and the impairment of spermatogenesis^(13), (14), (15), (16)^. Sensory, sympathetic, parasympathetic, and motor neurons are affected in these mice to varying degrees. Moreover, mice with an introduced mutation at tyrosine 1062 in RET, whose phosphorylation by GDNF stimulation is crucial for RET downstream signaling, such as the RAS-MAPK and PI3K-AKT pathways, showed phenotypes similar to, but milder than, RET KO mice ^(17), (18)^. These findings confirm the biological importance of GDNF/GFRα1/RET complex formation and resultant signaling activation during morphogenesis.

Similarly, mice that lack NRTN or GFRα2 have similar defects in enteric and parasympathetic innervations ^(19), (20)^. The parasympathetic cholinergic innervation is dramatically reduced in the lacrimal and submandibular salivary glands as well as in the intestines. However, NRTN-deficient mice grow normally, whereas GFRα2-deficient mice show growth retardation. GFRα3 KO mice exhibit severe defects in the superior cervical ganglion but not in other ganglia ^(21)^. Therefore, each GFL-GFRα-RET complex has been found to have specific roles in vivo.

## *RET* Fusion in Human Cancers

To date, *RET *fusion with other partner genes has been reported in a variety of human cancers, including papillary thyroid carcinoma (PTC) ^(22), (23), (24)^ and non-small cell lung cancers (NSCLCs) ^(25), (26), (27)^. *RET *fusion has been detected in 5%–35% of adult PTCs, in which rearrangement with the *CCDC6 *gene has most frequently been observed (named *RET/PTC1*, Figure 1). *RET *and *CCDC6 *genes are located on the long arm of chromosome 10, and this gene fusion is induced by intrachromosomal inversion. The other 5′ partner genes for *RET *fusion in PTC include *PRKAR1A*, *NCOA4*, *GOLGA5*, *TRIM24*, *TRIM33*, *KTN1*, and *RFG9* ^(24)^. These genes, except for *NCOA4*, which is located on chromosome 10, generate fusions with *RET *by interchromosomal translocation. After the Chernobyl nuclear reactor accident occurred in 1986, the occurrence of childhood PTC in contaminated regions dramatically increased, reaching approximately 10-fold increase 10 years after the accident. Of the childhood cases, ~60% to ~70% displayed *RET *rearrangement with 5′ fragment of the *NCOA4* gene (named *RET/PTC3*, Figure 1), and this fusion, like the *CCDC6-RET *fusion, was induced by the paracentric inversion of chromosome 10 ^(23), (28)^.

Breakpoints preferentially occur in restricted regions of *RET*, most commonly within intron 11^(24)^. This results in fusions, including in the cytoplasmic kinase domain of RET. Less frequent breakpoints involve introns 7 and 10, leading to the inclusion of the RET transmembrane domain. The 5′ partner genes contain distinct putative dimerization domains, such as a coiled coil domain, which mediates the ligand-independent activation (dimerization) of chimeric RET oncoproteins.

*RET *fusion has also been identified in 1%–2% of NSCLCs ^(25), (26), (27)^, with *KIF5B-RET *being the most commonly identified (Figure 1). *CCDC6*,* NCOA4*, and *TRIM33 *are also partner 5′ genes for *RET *fusion in NSCLCs ^(24)^. *KIF5B* is located on the short arm of chromosome 10, and *KIF5B-RET *fusion is created via pericentric inversion.

The next-generation DNA and/or RNA sequencing approach has led to the identification of *RET *fusion in a variety of cancers, including colorectal, breast, ovarian, and salivary gland cancers, as well as Spitz tumors and chronic myeloproliferative neoplasms ^(29), (30), (31)^. Large-scale analyses have reported that *RET *fusion can be detected in 0.2% of colorectal cancers (6/3117 cases) ^(32)^ and 0.1% of breast cancers (8/9693 cases) ^(33)^. *CCDC6-RET *and *NCOA4-RET* fusions have been identified in both cancers. Interestingly, a high frequency of *RET *fusion (42.4%), including *NCOA4-RET* and *TRIM27-RET*,**has been detected in salivary intraductal carcinomas, suggesting a role of *RET *fusion in the development of a particular type of salivary gland cancer ^(34)^.

## *RET* Mutations in Hereditary and Non-hereditary Cancers

Germline *RET *mutations are responsible for the development of autosomal dominant multiple endocrine neoplasia types 2A and 2B (MEN2A and MEN2B) and familial medullary thyroid carcinoma (FMTC) ^(35), (36), (37)^, all of which confer an increased risk (~100%) of developing medullary thyroid carcinoma (MTC). MEN2A is characterized by the presence of MTC combined with the development of pheochromocytoma and parathyroid hyperplasia/adenoma in ~50% and ~20% of affected family members, respectively. Moreover, lichen amyloidosis is occasionally observed. MEN2B is characterized by the development of MTC with an early age of tumor onset and displays a more complex phenotype, including pheochromocytoma, mucosal neuroma, ganglioneuromatosis of the intestine, thickening of corneal nerves, and marfanoid habitus. FMTC only develops MTC in families, usually in the later stage of life.

**Figure 2.**
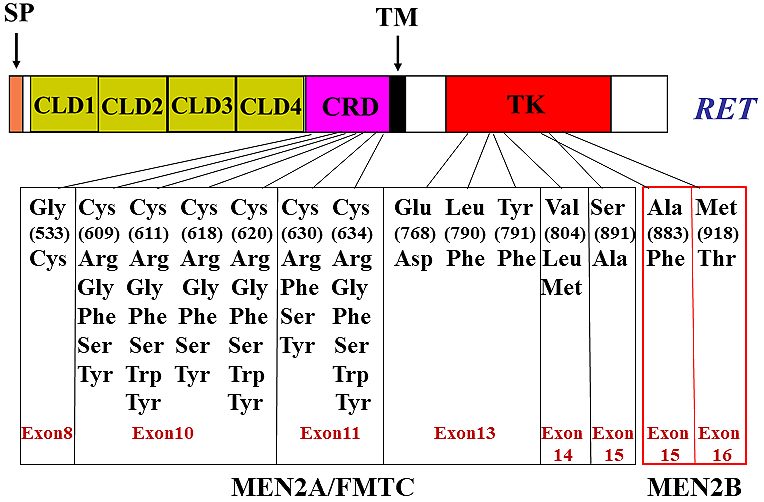
*RET* missense mutations were identified in multiple endocrine neoplasia 2A (MEN2A) and 2B (MEN2B) and familial medullary thyroid carcinoma (FMTC).

*MEN2A* mutations in the majority of families (~95%) have been identified in one of six cysteine residues (codons 609, 611, 618, and 620 in exon 10, and codons 630 and 634 in exon 11) in the cysteine-rich region of the RET extracellular domain (Figure 2). Of these cysteine mutations, missense mutations at codon 634 have been identified in ~85% of the affected MEN2A family members ^(38)^. We and Santoro et al. previously elucidated the molecular mechanism of RET activation by cysteine mutations. When a cysteine residue is substituted with a non-cysteine residue, a partner cysteine that is normally involved in the formation of an intramolecular disulfide bond is freed and forms an aberrant intermolecular covalent disulfide bond between two mutant RET, leading to its dimerization and activation ^(39), (40)^.

Cysteine mutations in the RET extracellular domain also cause the FMTC phenotype. Interestingly, contrary to MEN2A families, a higher frequency (~60%) of non-cysteine 634 substitutions (cysteines 609, 611, 618, 620, and 630) and a lower frequency (~30%) of cysteine 634 substitutions have been detected in FMTC families ^(41)^. We found that the transforming activities of RET with cysteine 609, 611, 618, or 620 mutations were considerably lower than those of RET with a cysteine 634 mutation because of the impairment of cell surface expression of the former four cysteine mutants ^(42)^. The low transforming activity may predispose to the development of FMTC rather than MEN2A. Moreover, Gly533Cys (G533C) (exon 8 in the RET extracellular domain) ^(43)^, Glu768Asp (E768D), Leu790Phe (L790F), Tyr791Phe (Y791F), Val804Met/Leu (V804M/L), and Ser891Ala (S891A) substitution (exons 13–15 in the RET kinase domain) ^(24)^ have been reported in some families of MEN2A and/or FMTC (Figure 2).

Two missense mutations, Met918Thr (M918T) (exon 16) and Ala883Phe (A883F) (exon 15), in the kinase domain are associated with MEN2B (Figure 2). Of the MEN2B patients, ~95% carry the M918T mutation and fewer than 4% carry the A883F mutation ^(44)^. The M918T mutation increases the kinase activity as monomers and the presentation of substrates for trans-autophosphorylation ^(40)^, which is conferred by conformational changes in the activation loop of the kinase domain. However, the mechanisms by which the different clinical phenotypes between MEN2A and MEN2B develop remain elusive.

According to data published in a public database in 2015 (Catalog of Somatic Mutations in Cancer), somatic *RET *mutations have been detected in 677 of 1662 (41%) samples from patients with MTC. M918T mutations were most frequent in these patients, and cysteine mutations (C634, C630, C618, and C611) as well as E768D, V804M/L, and A883F mutations were observed in sporadic MTCs ^(24)^. Oncogenic *RET *mutations were also detected in 0.5% (8/1489) of colorectal cancer and 0.2% (16/9693) of breast cancer, including Glu511Lys (E511K), G533C, Cys634Arg (C634R), E768D, V804M, and M918T mutations ^(32), (33), (45)^. Moreover, the increasing use of NGS has uncovered *RET *mutations in a variety of cancers, including endometrial and ovarian cancers, hepatomas, skin melanomas, glioblastoma multiforme, meningiomas, gastrointestinal stromal tumors, Merkel cell carcinomas, paragangliomas, and atypical lung carcinoids ^(29)^.

## Loss of Function *RET* Mutations in Non-malignant Diseases

**Figure 3.**
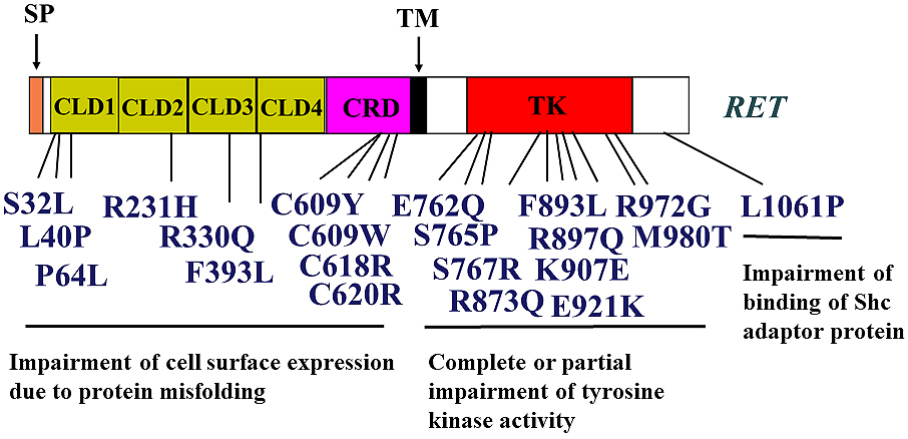
*RET *missense mutations identified in Hirschsprung’s disease.

Hirschsprung’s disease (HSCR) is a congenital malformation associated with aganglionosis of the gastrointestinal tract. *RET *mutations account for *~*50% of familial and 10%–20% of sporadic cases of HSCR ^(46), (47), (48), (49)^. A variety of missense, nonsense, and frameshift mutations have been identified along the entire coding sequence of *RET* (Figure 3)*. *Moreover, partial or complete deletion of one allele of *RET* has been reported in some HSCR cases. Furthermore, mutations in non-coding sequences, including the promoter/enhancer regions, have been detected in HSCR, resulting in the alteration of the regulation of *RET *expression ^(50)^. Interestingly, the penetrance of the mutant allele in familial HSCR is low and is significantly higher in males than in females, suggesting the existence of one or more modifier genes in HSCR.

We and others have performed functional analyses of missense mutations in the RET coding region identified in patients with HSCR. Based on these studies, four mechanisms appear to be responsible for HSCR development (Figure 3). (1) Most mutations in the RET extracellular domain impair its cell surface expression, most likely because of misfolding of the RET protein ^(42), (51), (52), (53)^. Low levels of RET on the cell surface cannot transmit sufficient signals to elicit GDNF-mediated biological activities in enteric neuroblasts during embryogenesis. (2) Mutations in the kinase domain that occur at highly conserved amino acids among receptor tyrosine kinase families almost completely abolish RET kinase activity ^(54), (55), (56)^. (3) Mutations in the kinase domain (e.g., Glu762Gln (E762Q), Ser767Arg (S767R), Arg972Gly (R972G), and Met980Thr (M980T)) markedly impair the activity of PLCγ, whereas RAS and PI3K pathways are mildly affected ^(56)^. (4) Mutations in the carboxyl-terminal tail impair intracellular signaling because of decreased SHC binding to RET ^(57), (58), (59)^.

Moreover, *RET *mutations are less commonly associated with congenital abnormalities of the kidney and urinary tract and congenital central hypoventilation syndrome (CCHS), also known as “Ondine’s Curse” ^(60)^. In some cases, the CCHS presents together with the HSCR.

## Therapeutic Application of Kinase Inhibitors to RET-Altered Cancer

A variety of multikinase inhibitors with activity against RET have been developed and are available to treat RET-altered cancers. These include cabozantinib, vandetanib, lenvatinib, ponatinib, sorafenib, sunitinib, alectinib, regorafenib, and RXDX-105, which are classified into distinct groups by their mode of kinase inhibition ^(60)^. For example, vandetanib and sunitinib are type I inhibitors that bind within the adenosine 5′-triphosphate (ATP)-binding pocket in the active conformation of the RET kinase ^(61)^. Cabozantinib, sorafenib, ponatinib, and RXDX-105 are type II inhibitors that bind within the ATP-binding pocket in the inactive conformation of the RET kinase ^(61), (62), (63)^. However, the clinical efficacy of multikinase inhibitors in RET-altered patients appears limited (lower overall objective response ratios (ORRs) and shorter progression-free survival) and has significant off-target toxicities ^(60)^. This is most likely because these inhibitors are not specifically designed for targeting RET, and their effects were initially focused on other kinases, including VEGFR, EGFR, MET, and ALK. Cabozantinib, vandetanib, and lenvatinib more potently inhibit VEGFR2, despite the RET kinase domain having significant homology to that of VEGFR2.

Secondary *RET *mutations that confer the resistance of RET-altered cancers to multikinase inhibitors during therapy have been reported. V804L and V804M are representative examples that alter the valine residue in the gatekeeper site of RET ^(64), (65)^. These mutations are thought to introduce steric clashes between the leucine and methionine side chains and the 4-bromo-2-fluorophenyl group of vandetanib. I788N is another secondary mutation known as the non-gatekeeper mutation ^(62)^.

Recently, RET-specific kinase inhibitors LOXO-292 and BLU-667 have been developed to inhibit the kinase activity of both wild-type RET and the gatekeeper mutant RET ^(66), (67), (68)^. ORR (>50%) and the duration of response (>6 months) using these inhibitors showed greater benefit for RET-altered patients in clinical trials. Intrinsic resistance to multikinase inhibitors occurs in cancers that carry the *KIF5B-RET *fusion gene. Notably, the intrinsic resistance conferred by this fusion has been overcome with treatment of LOXO-292 and BLU-667, stressing the necessity for the development of RET-specific inhibitors.

## Conclusions

*RET* was first identified 35 years ago, and its oncogenic rearrangements and mutations have been reported in a variety of cancers, including thyroid cancer, NSCLC, MEN2A, and MEN2B. Recent developments in RET-specific kinase inhibitors are making progress in providing considerable benefit to patients with RET-altered cancer. Moreover, studies regarding GDNF-RET signaling continue to have a profound impact on neuroscience and developmental biology. Based on the efficacy of GDNF as a survival factor of dopaminergic and motor neurons, establishing neurons expressing GDNF from iPS or ES cells could be beneficial for treating neurodegenerative diseases, such as Parkinson’s disease. Future studies regarding RET functions will further contribute to a deeper understanding of the molecular mechanisms of the enteric nervous system and kidney development as well as of spermatogenesis.

## Article Information

### 

This article is based on the study, which received the Medical Award of The Japan Medical Association in 2019.

### Conflicts of Interest

None

### Disclaimer

Masahide Takahashi is one of the Consultants of JMA Journal and on the journal's Editorial Staff. He was not involved in the editorial evaluation or decision to accept this article for publication at all.
